# Editorial: Effects of Driver Support Systems on Cognitive and Affective Processes Underlying Driving Behaviors

**DOI:** 10.3389/fpsyg.2021.765838

**Published:** 2021-10-25

**Authors:** Mariaelena Tagliabue, Amanda Stephens, Tova Rosenbloom, Mark Sullman, Massimiliano Gastaldi

**Affiliations:** ^1^Department of General Psychology, University of Padua, Padua, Italy; ^2^Department of Civil, Environmental and Architectural Engineering, University of Padua, Padua, Italy; ^3^Mobility and Behavior Research Center—MoBe, University of Padua, Padua, Italy; ^4^Accident Research Centre, Monash University, Clayton, VIC, Australia; ^5^Department of Management, Bar Ilan University, Ramat Gan, Israel; ^6^Department of Social Sciences, University of Nicosia, Nicosia, Cyprus

**Keywords:** ADAS, automation, mindless regulation, fitness to drive, multitasking, sense of agency

Driving is a challenging task which involves complex interactions between psychological factors that can underpin whether on-road behaviors are safe or not. The knowledge of these factors is crucial to achieve targeted reductions in road trauma set by governments and regulators around the world. Advances in vehicle technology is among one of the strategies expected to improve road safety. Recently, much research has been conducted to develop these applications and investigate their potential usefulness, taking into account the characteristics and limitations of the human cognitive/emotional/motivational system. Over the past few decades, the development of automation and connected autonomous vehicles has provided the tools for on-road mobility which is less prone to human errors, and basic research aimed at investigating the impact of these vehicles on the users, to identify the appropriate guidelines, has become increasingly needed. With this in mind, this special issue focused on contributing to the wealth of knowledge and debate in the field of road safety, in particular by tackling some key issues around driver support systems, cognitions and affect.

Considering that cognition is affected by emotional processes, emotional control plays a crucial role in complex behaviors. Consequently, negative emotions, such as stress, may have detrimental effects on driving performance. This issue was discussed by Béquet et al. in their systematic review, which focused on mindless technologies (which promote changes by acting without involving conscious awareness, thus avoiding performance impairment) for stress regulation in driving contexts. These researchers illustrated how much such technologies can improve safety and comfort during driving tasks. The review combined information from patents and research, highlighting the need to better support technical applications, which often lack a robust theoretical background, with research data from both laboratory and ecological settings. The study by Tinella et al., represents an essential step for applied research and helps to fill this gap by demonstrating the contribution of sociodemographic (age and gender) and cognitive (spatial mental transformation skills) variables to stress resilience. This may inspire developers and the automotive industry to identify solutions suitable for an increasingly large part of the population. As suggested by the authors, the use of in-vehicle cameras and monitors aimed at supporting the driver in navigation and visual exploration by expanding the visual field, may interfere with the spatial mental transformation involved in driving. This attributes a key role to the detailed knowledge of such mental skills.

Lajunen and Sullman, provided an important contribution related to the possible usefulness of autonomous vehicles in supporting elderly driver's limits/difficulties. Age plays a crucial role in reducing fitness to drive, as confirmed by Tinella et al.'s results. However, the effectiveness of such technologies also depends on attitudes of elderly toward them, and Lajunen and Sullman's study showed that the elderly's propensity to adopt autonomous vehicles decreases as the level of automation increases. This could reduce the spread of such technologies among these kinds of road users, and therefore, also their effectiveness in improving road safety and mobility. If politicians plan interventions for promoting the use of autonomous vehicles among the elderly, these campaigns should focus on improving the acceptance of autonomous vehicles.

In line with Béquet et al.'s claim that technology can also have a detrimental effect, if users experience a decrease in feelings of control, Wen et al. focused on a side effect of these tools, namely the decreased sense of agency. This occurs when the assistance systems reduce the driver's degree of engagement. Sense of agency (the feeling of control over external events) tends to decrease as the assistance system gets involved, which could reduce user acceptance of the assistance system (and, therefore, the propensity to employ it). Wen et al. demonstrated that, during simulated driving, the more the effects of the assistance system are consistent with the driver's intentions, the less the sense of agency is reduced, the more the driver remains engaged in the task and his/her performance improves in comparison with conditions in which the system is not activated. The authors consistently recommend that assistance technologies be developed which avoid conflicting with the user's intentions, as this would preserve the driver's sense of agency and allow an improvement in driving behavior.

Tagliabue et al., tested a proactive feedback system which informs the drivers about speeding during simulated moped driving. They showed that the effectiveness of the feedback may persist over time, despite being induced with a short training, but the observed effect varied depending on the driving style. People with an aggressive driving style seem to benefit to a greater extent from the alert delivered. Moreover, on the one hand the introduction of feedback could improve safe driving, while on the other too many in-vehicle signals may have a detrimental effect, because alert stimuli require responses, thus creating multitasking conditions which increase the risk of fatal distractions. In this view, studies like that of Fraschetti et al., showing that attitudes toward multitasking differ depending on the gender and are modulated by psychological characteristics such as subjective self-efficacy, risk perception and sensation seeking, may provide interesting clues to conceive interventions aimed at reducing inappropriate behaviors, such as using the mobile, or to tailor the support system to the targeted category of drivers.

All contributions to the present Research Topic converge in showing that automation by itself is not enough to reduce road trauma, since its degree of acceptance among users will determine the choice to use them or not, thus modulating its actual effectiveness on road safety. A deep knowledge of the cognitive, emotional, and motivational aspects involved in driving will provide insights to foster the spread of driver support systems.

## Author Contributions

All authors listed have made a substantial, direct and intellectual contribution to the work, and approved it for publication.

## Conflict of Interest

The authors declare that the research was conducted in the absence of any commercial or financial relationships that could be construed as a potential conflict of interest.

## Publisher's Note

All claims expressed in this article are solely those of the authors and do not necessarily represent those of their affiliated organizations, or those of the publisher, the editors and the reviewers. Any product that may be evaluated in this article, or claim that may be made by its manufacturer, is not guaranteed or endorsed by the publisher.

